# The Haptic Recognition of Geometrical Shapes in Congenitally Blind and Blindfolded Adolescents: Is There a Haptic Prototype Effect?

**DOI:** 10.1371/journal.pone.0040251

**Published:** 2012-06-28

**Authors:** Anne Theurel, Stéphanie Frileux, Yvette Hatwell, Edouard Gentaz

**Affiliations:** 1 Laboratoire de Psychologie et NeuroCognition (UMR CNRS 5105), Université Pierre Mendès-France, Grenoble, France; 2 Institut National Supérieur de Formation et de Recherche pour l'Education des Jeunes Handicapés et les Enseignements Adaptés (INS HEA), Suresnes, France; University of Bologna, Italy

## Abstract

**Background:**

It has been shown that visual geometrical shape categories (rectangle and triangle) are graded structures organized around a prototype as demonstrated by perception and production tasks in adults as well as in children. The visual prototypical shapes are better recognized than other exemplars of the categories. Their existence could emerge from early exposure to these prototypical shapes that are present in our visual environment. The present study examined the role of visual experience in the existence of prototypical shapes by comparing the haptic recognition of geometrical shapes in congenitally blind and blindfolded adolescents.

**Methodology/Principal Findings:**

To determine whether the existence of a prototype effect (higher recognition of prototypical shapes than non prototypical shapes) depended on visual experience, congenitally blind and blindfolded sighted adolescents were asked to recognize in the haptic modality three categories of correct shapes (square, rectangle, triangle) varying in orientation (prototypical/canonical orientation vs. non prototypical/canonical orientation rotated by 45°) among a set of other shapes. A haptic prototype effect was found in the blindfolded sighted whereas no difference between prototypical and non prototypical correct shapes was observed in the congenitally blind. A control experiment using a similar visual recognition task confirmed the existence of a visual prototype effect in a group of sighted adolescents.

**Conclusion/Significance:**

These findings show that the prototype effect is not intrinsic to the haptic modality but depends on visual experience. This suggests that the occurrence of visual and haptic prototypical shapes in the recognition of geometrical shape seems to depend on visual exposure to these prototypical shapes existing in our environment.

## Introduction

Geometrical shapes (rectangle, triangle, etc.) can be considered as categories including an infinite number of particular shapes that share common properties [1]. Even though adults and children are able to categorize visual shapes correctly in some conditions according to abstract geometrical rules, they show a bias toward prototypical shapes both in perception (judgment of the typicality of visually presented triangles and quadrilaterals) and production tasks (drawing a number of different exemplars of the category) [2]. Thus, shape categories tended to be graded structures organized respectively around a prototype as it was already observed in other categories [2]–[6]. However, the study of Feldman [2] did not investigate adult's prototypes for finer shape categories (e.g. rectangle) and children's prototypes, nor did it analyze whether the drawings and judgments of typicality showed a bias toward a preferred orientation since the descriptors were invariant across rotation. Recently, these limits were addressed in examining the production of one rectangle and one triangle in adults and children [7]. Results showed that both populations tended to draw shapes characterized by their ratio between the sides and aligned with the edge of the table (horizontal orientation). These findings generalized those of Gentaz and his colleagues [8]–[10] who showed that at the age of 5, the recognition of rectangles or triangles among other shapes is better for some particular shapes in each category. In summary, these results indicated that in adults and children, in both the perception and production domains, shape categories tend to be graded structures organized around a prototype in which the horizontal orientation plays an important role in their definition.

However, the origins of the bias toward prototypical shape are still in debate. Feldman, (2000, p. 164) [2] proposed that *“these subjective shape distributions originate not in simple empirical observation but rather in a nexus of subtle mental stereotypes about regularity of form and pattern”*. The findings of Gentaz and his colleagues [8]–[10] are partially in line with this hypothesis. They may suggest that prototypical shapes emerge from children's early visual exposure to rectangular and triangular shapes that are present in their environment, where the shapes corresponding to adults' prototypes could be more frequent (in artworks, toys, etc.).

Consequently, the question was to determine whether the presence of prototypical shapes holds true for objects encoded in the haptic modality. This question was not trivial because of several specificities of haptic perception [11]. Indeed, to compensate for the small object contact area and to perceive whole objects, manual haptic perception requires several voluntary exploration movements (labeled “Exploratory Procedures”), varying according to the characteristics of what is to be perceived [12]–[14]. Some authors contend that this results in a fragmented apprehension, sometimes partial and always very sequential, which overloads working memory and requires, at the end of exploration, a mental integration and synthesis to lead to a unified object representation [11], [15]. Given the differences between visual and haptic encoding, Woods, More and Newell (2008) [16] investigated in blindfolded adults whether canonical (or prototypical) views also exist in haptic (familiar and novel) object recognition, as it is the case in vision [17]–[19]. Results revealed the occurrence of preferred orientations which promoted better accuracy, consistent across participants. Authors minimized the possibility that visual knowledge or imagery played a role in the choice of preferred views in haptic perception by choosing both familiar and novel objects. Nevertheless, there were some cases where participants oriented the objects to provide a better view of the most informative surface to the mind's eye. This suggests that visual experience influenced canonical views through touch. For example, the canonical view was typically such that the most informative surface of the object faced the observer rather than faced away and this is more conducive to efficient visual, rather than haptic object recognition [18]. Indeed, the back side of a hand sized object is more accessible than the front for the haptic system (when an object is haptically explored, fingers feel the back of object whereas only the thumb contact the front) whereas the front is more accessible than the back to the visual system.

Therefore, the comparison between congenitally blind and blindfolded sighted adolescents could allow us an evaluation of the role of visual experience in the manifestation of the prototype effect. In the latter populations, the haptic modality is coordinated with the visual modality whereas in the former one, it is not. Lederman et al. (1990) [20] argued that during the recognition of tactile drawings, the subjects adopt the visual mediation model, in which the haptic data are translated into visual pictures, which are then processed by the visual system. On the other hand, because congenitally blind adolescents are highly trained in haptics, they may rely on specific haptic perceptual cues modifying the relative difficulty of the different shapes tested. Some studies showed that blind subjects performed as good or better than sighted ones in tasks such as size discrimination with a cane [21], haptic object exploration and recognition [22], and tactile recognition of 2D angles and gratings [23], whereas other studies showed that blind subjects were impaired in tasks such as haptic orientation discrimination [24], spatial imagination [25], and mental rotation [26]. These studies highlighted that according the task, two factors act in different directions: visual representations and visual recoding may induce similar performances in blindfolded sighted and sighted people whereas lack of vision and intensive training in haptics may induce specific performance in congenitally blind adolescents.

In the present research, we investigated therefore with the same haptic recognition task whether the “prototype effect” exists in haptics by testing a group of congenitally blind adolescents and a group of blindfolded sighted adolescents. The “prototype effect” corresponds to the better recognition of prototypical shapes than non prototypical shapes and occurs independently of the rate of global performances. This means that it may be present in relatively accurate performances as well as in poor ones. Each participant was asked to recognize with their haptic modality the category of correct shapes (square, rectangle, triangle) varying in orientation (prototypical vs. non prototypical) among a set of other geometrical shapes varying in angle between sides and in number of side. Our aim was to determine whether the performances and manual exploratory strategies observed in the congenitally blind would be different from those found in blindfolded sighted adolescents. To analyze the performances, the recognition time (seconds) and the number of correct responses were measured. In order to ensure that the differences on performances observed between the two groups were not due to different haptic strategies, the manual exploratory procedures used by participants during the recognition of each shape stimuli were analyzed. If visual experience was not involved in the existence of prototypical shape, the same pattern of results (an intrinsic haptic prototype effect or no haptic prototype effect) should be observed in both populations. By contrast, if the prototype effect was in some way dependent on visual experience, no haptic prototype effect should be observed in the congenitally blind adolescents, whereas this effect would be present in the same tasks and the same conditions in blindfolded sighted adolescents.

## Methods

### 1. Participants

Fourteen congenitally blind (three girls and eleven boys) without associated disorders, aged 15.5 years on average (SD = 3.16) integrated in ordinary schools in France but receiving special support from teachers for visually impaired participated in this study. None of the blind subjects having light perception could discriminate shape or hand movements. Table 1 shows the characteristics of these congenitally blind adolescents. These participants were matched on mean age and educational level with fourteen sighted (two girls and twelve boys) aged 15.5 years on average (SD = 0.47) schooled in a French technical high school. The present study was conducted in accordance with the Declaration of Helsinki. It was conducted with the understanding and the written consent of each participant or a parent for underage which was obtained. It was approved by the local ethic committee of the LPNC (CNRS and University of Grenoble) and in accordance with the ethic convention between the academic organization (LPNC-CNRS) and educational organizations for blind people.

**Table 1:** Characteristics of congenitally blind who participated in the experiment.

Participant	Age	Sex	Cause of the deficiency
1	11	F	Retinitis pigmentosa
2	12	F	Leber's amaurosis
3	12	M	Optic nerve atrophy
4	16	F	Glaucoma
5	15	M	Microphtalmia
6	18	M	Microphtalmia
7	18	M	Leber's amaurosis
8	17	M	Retinitis pigmentosa, Leber's amaurosis aaamauamaamaurosis
9	18	M	Retinitis pigmentosa
10	18	M	Unspecified
11	9	M	Congenital cataract
12	18	M	Unspecified
13	18	M	Microphtalmia
14	17	M	Unspecified

### 2. Stimuli

Three categories of geometric shapes were presented to subjects: square, rectangle and triangle. For each category of shape, there was a correct shape and two distorted shapes, each varying from the correct shape according to a criterion: the angle between sides (changing the degree of the angle between sides of the correct shape) or the distortion of the base (distort the base in order to change the shape into a polygon with an interior angle of 140°). Concerning the change in the degree of the angle, the four right angles (90°) of squares and rectangles have been modified in two obtuse angle of 105° (the top right and the left bottom angle of the shape) and in two acute angle of 75° (the top left and the right bottom angle). For triangles the three angles of 60° have been modified in three angles of 20°(left bottom angle), 30° (right bottom angle) and 130°(top angle). Stimuli consisted of embossed geometric shapes cut in foam of 4 mm thick. As some authors had observed that picture size influences recognition rate [27]–[28] all correct and distorted shapes were presented in small and large sizes. The sizes of the large shapes were 5 cm for the square, 5 cm×7 cm for the rectangle and 5 cm side and base and 4.33 cm height for the triangle. The sizes of the small shapes were 2.5 cm for the square, 2.5 cm×3.5 cm for the rectangle and 2.5 cm side and base and 2.2 cm height for the triangle. Each embossed shape was fixed to a hexagonal board (20 cm in diameter) in order to give a systematic horizontal baseline available for each shape.

**Figure 1:**
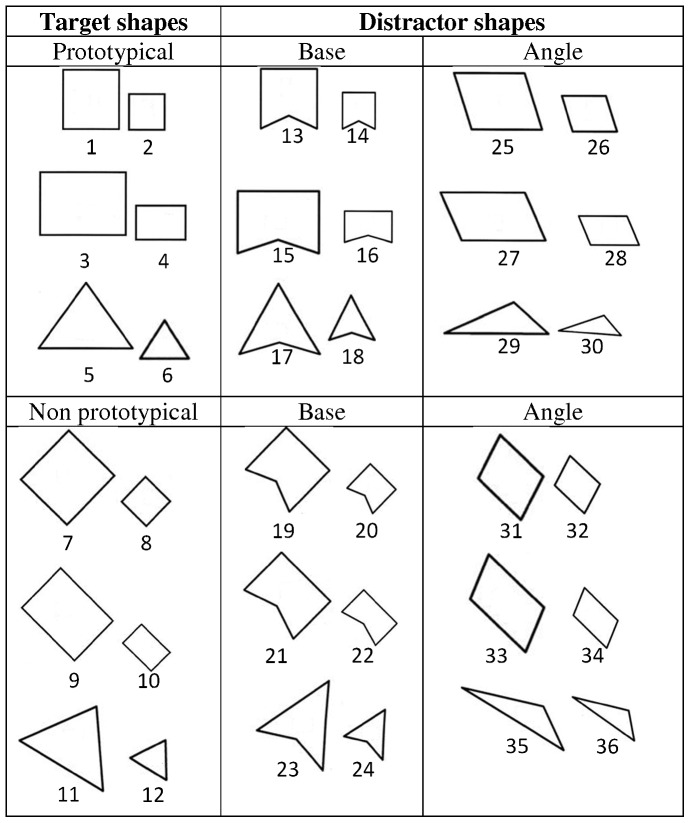
Thirty-six geometrical shapes presented to congenitally blind and blindfolded sighted adolescents.

For each category of geometric shapes, correct and distorted shapes (small and large) were presented in a canonical way (i.e., the shape presented standing on its base) or in an oriented way (i.e., the canonical shape was rotated by 45 degrees). The correct shapes presented on the canonical way were called «prototypical shape» and those presented in an oriented way were called “non prototypical shape”. For each category of geometric shapes (square, rectangle, triangle), there were twelve shapes: one correct shape and two distorted shapes, each presented in two size format (small, large) and in two presentation format (prototypical, non prototypical) (Figure 1). In sum, thirty-six stimuli were presented to the participants.

To examine the relevance of the set of stimuli used in this main haptic experiment, we carried a control experiment (Experiment S1) in order to confirm the existence of “the prototype effect” in sighted adolescents matched on age and educational level in using similar visual stimuli. The results confirmed that the prototypical correct shapes were better (rate in %) and faster (reaction time in ms) recognized than the non prototypical correct shapes.

### 3. Experimental condition and procedure

Participants performed a haptic recognition task of geometrical shapes. The experimenter presented one by one the different shapes (correct and distorted, in random order) to the participant whose task was to correctly identify (with no time limit) by touch embossed geometric shapes (square, rectangle or triangle). The non-dominant hand held the support of the shape on the table and the other dominant hand explored the shape. The sighted participants worked blindfolded by a mask. The shapes were randomly presented. The instruction was: “I'm going to show you embossed geometric shapes and you will have to touch them. Then, you have to tell me whether you think the shape that you touched is a square, rectangle, triangle or you'll say “none” if you think it is none of these three shapes”. No feedback on responses and no instructions on how to explore were given to participants.

The whole experiment was videotaped in order to measure the recognition time of each stimulus and the manual exploratory procedure used by each participant. To analyze the performances, the nature of responses (correct or false recognition) and the recognition time (seconds) were measured. Regarding the nature of responses, we considered as correct recognition when a subject identified a square for the shapes 1-2-7-8, a rectangle for the shapes 3-4-9-10 and a triangle for the shapes 5-6-11-12-29-30-35-36 (cf. Figure 1) and as false recognition when a subject identified wrongly a square, a rectangle or a triangle during the presentation of the other shapes not listed above. Recognition time corresponded to the period between the first contact of the subject hand with the item and when he gives a correct answer. We investigated the manual exploratory procedures used by participants during the recognition in order to ensure that the differences in recognition times observed between the two groups are not due to different haptic strategies. According to the coding used by Lederman and Klatzky (1987) [12] and on the metric procedures described by Appelle, Gravetter & Davidson (1980) [29], three exploratory procedures were identified (EP): (1) The enclosure (E) is defined by any movement involving several fingers grasping the embossed shape; (2) the contour following (CF) is defined by moving a single finger or two fingers side by side on the turn of the figure; and (3) the metric procedure (M) is defined by the use of fingers like a pinch (thumb and index) to assess the distance between the two fingers. We noted for each geometric shape the number of times it was recognized by the participants with the different exploratory procedures.

## Results

### 1. Analysis of performances

Preliminary ANOVAs on performances with visual status as intersubject factor, shape and size as intrasubject factor and age as continuous predictor revealed that the age factor and size factor were not significant and did not interact with other factors [all F<1]. Consequently, 2 (visual status)×2 (shape) ANOVAs were carried out on recognition rates and recognition times for correct responses considering the visual status factor (Congenitally Blind, Sighted) as intersubject and the shape factor (Prototypical, Non prototypical) as intrasubject.

**Figure 2:**
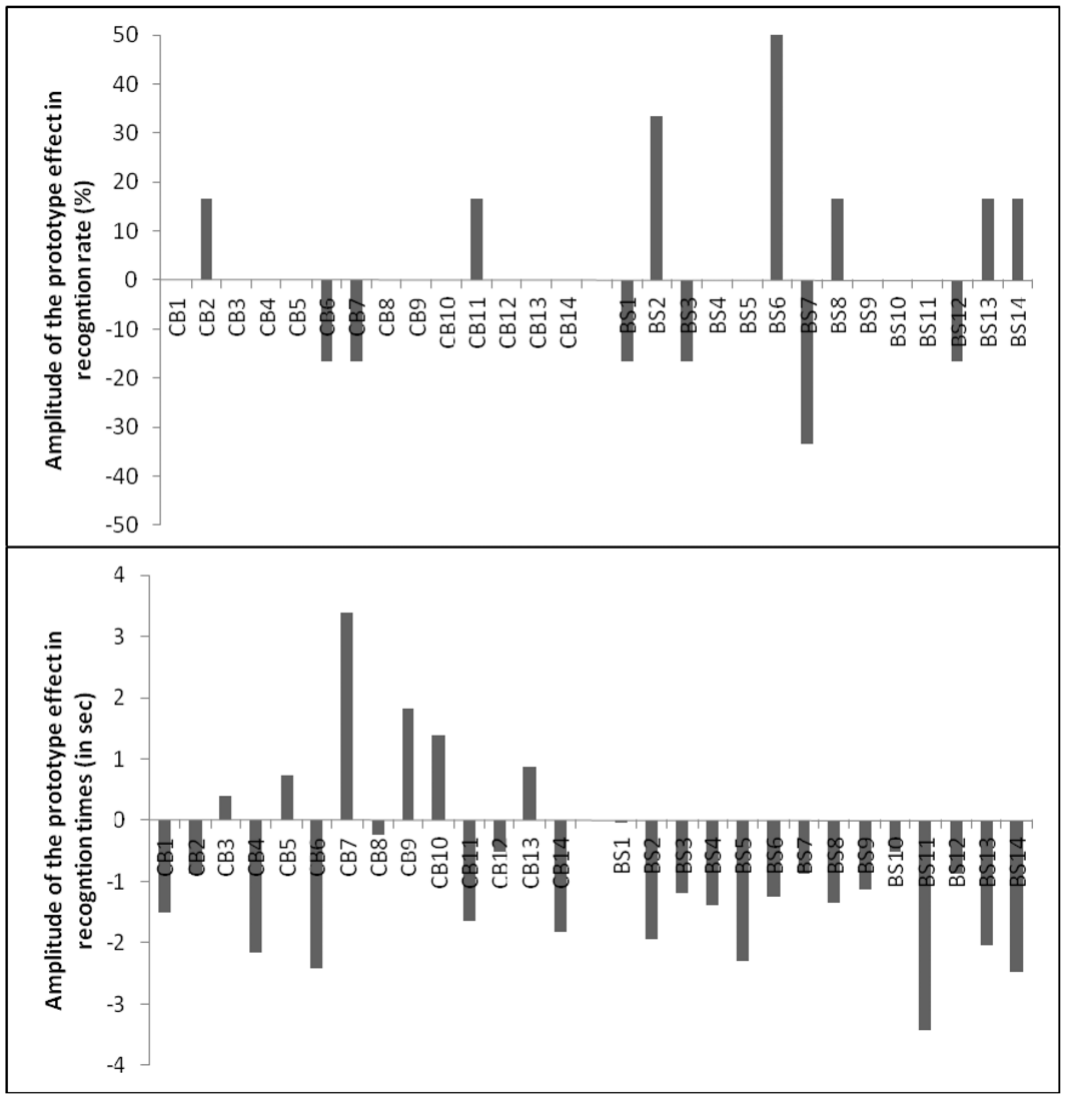
The amplitudes of the prototype effect for each subjects' performances (CB: congenital blind and BS: blindfolded sighted). The amplitudes in the recognition rates and in the recognition times correspond to the difference between the prototypical correct shapes and the non prototypical shapes: amplitude of zero means an absence of prototype effect and a negative amplitude means an occurrence of prototype effect.

Table 2 shows the mean recognition times and recognition rates of the correct shapes as a function of visual status and shape for the three categories of shapes (Table 2). Because the “prototype effect” corresponds to the better and faster recognition of prototypical shapes than non prototypical shapes, Figure 2 shows the amplitudes of the prototype effect for each subject's performances. The amplitude in the recognition rates and in the recognition times corresponds to the difference between the prototypical correct shapes and the non prototypical shapes: an amplitude of zero means an absence of prototype effect and a negative amplitude means an occurrence of prototype effect.

**Table 2:** Mean recognition times (s) and rates (%) (and SD) of the target shapes as a function of visual status and shape.

		Time in s	Rate in %
Congenitally Blind	Prototypical	5.06 (2.32)	96.43 (7.10)
	Non prototypical	5.24 (1.96)	96.43(9.65)
	Total	5.15 (2.11)	96.43 (8.31)
Blindfolded Sighted	Prototypical	5.40 (1.26)	76.19 (19.30)
	Non prototypical	6.88 (1.74)	79.76 (14.88)
	Total	6.14 (1.67)	77.98 (17.00)

Regarding the recognition rates (in %), results showed a main effect of visual status [F(1,26) = 20.93; p<.01, with η^2^ = .45]: Correct shapes were better identified by the congenitally blind with a recognition rate of 96.43% (SD = 8.31) than by the sighted (M = 77.98%; SD = 17.00). The analysis did not reveal a main effect of shape [F(1,26)<1; p = .58]: The prototypical correct shapes (M = 86.31; SD = 17.60) were not better recognized than the non prototypical one (M = 88.09; SD = 14.95). Finally, the interaction between shape and visual status was not significant [F(1,26)<1; p = .58]. It should be noted that the change in the orientation of square did not lead the subjects to perceive it as a diamond in haptics (even if a square rotated 45 degrees is not a diamond since it still has 4 right angles). Indeed, the subjects equally recognized “diamonds” and “square on their bases” as correct square (74.11% and 76.79% respectively).

Regarding the recognition times (in sec.), results showed that the main effect of visual status was not significant [F(1,26) = 2.30; p = .14]. The analysis also revealed that the prototypical correct shapes (M = 5.15; SD = 2.11) were faster recognized than the non prototypical ones (M = 6.14; SD = 1.67) [F(1,26) = 10.62; p<.01, with η^2^ = .29]. Results showed a significant interaction between the visual status and shape factors [F(1,26) = 6.49; p<.05, with η^2^ = .20]. A HSD Tukey test revealed that while no difference was observed between prototypical correct shapes (M = 5.06 and SD = 2.32) and non prototypical correct shapes (M = 5.24 and SD = 1.96) in the congenitally blind (p = .96) the blindfolded sighted recognized the prototypical correct shapes (M = 5.40 and SD = 1.26) faster than the non prototypical one (M = 6.88 and SD = 1.74) (p<.01).

### 2. Analysis of manual exploratory procedures

Table 3 shows the rate of exploratory procedures used by participants as a function of their visual status and the nature of shape (Table 3). A 2 (visual status)×2 (shape) MANOVA was carried out on the rate of the three exploratory procedures with repeated measures on the first factor. Results revealed a main effect of visual status [Wilks (3,8) = 0.20; p<.01, with η^2^ = .80]. This means that the two groups did not use in the same manner the three exploratory procedures: blindfolded sighted used almost exclusively metric procedure whereas congenitally blind used also the other exploratory procedures. The main effect of shape was not significant [Wilks (3,8) = 0.574; p = .20] and the interaction between the visual status factor and the shape factor was not significant [Wilks (3,8) = 0.839; p = .68]. Finally the accuracy of the main exploratory procedure (metric) was examined as a function of group. In the congenitally blind, 7.03% of metric procedures were associated to an incorrect response versus 20.94% in the blindfolded sighted.

**Table 3:** Mean rate (%) (and SD) of the manual exploratory procedures as a function of visual status and shape.

	Congenitally Blind			Blindfolded Sighted		
	Exploratory procedure^1^ (Rate in %)			Exploratory procedure (Rate in %)		
	E	CF	M	E	CF	M
Prototypical	8.47 (5.35)	10.71 (9.85)	71.43 (11.07)	0 (0)	7.14 (9.04)	85.71 (4.52)
Non prototypical	14.29 (6.39)	10.98 (9.74)	71.43 (6.39)	2.38 (3.69)	7.14 (6.39)	82.14 (3.91)
Total	11.38 (6.39)	10.85 (9.34)	71.43 (8.61)	1.19 (2.78)	7.14 (7.46)	83.93 (4.44)

1 E = Enclosure; CF = Contour Following; M = Metric.

## Discussion

The objective of this study was to investigate the role of visual experience on the haptic recognition of geometrical shapes by congenitally blind and blindfolded sighted adolescents and in particular the occurrence of prototypical shapes. First, the recognition time analysis showed that the blindfolded sighted recognized prototypical shapes faster than non prototypical shapes while no difference was observed in the congenitally blind. Moreover, results showed that in the congenitally blind as in the blindfolded sighted, exploration procedures did not vary depending on the shape orientation. The differences observed between these two groups were thus not related to the characteristics of the exploratory movements of the participants. Nevertheless, the analysis of haptic recognition rates showed that both congenitally blind and blindfolded sighted recognized the prototypical shapes and the non prototypical ones in the same way. Yet, the measure of this recognition rate probably did not allow us to highlight such fine distinctions since the proposed task was not limited in time and was succeeded by participants at a very high level. More particularly, recognition rates of the congenitally blind were at ceiling. It could then be argued that the task was too simple for blind subjects whatever the shapes and that this factor could have masked the occurrence of the prototype effect on recognition time in congenitally blind. The fact that congenitally blind appeared more skilled than blindfolded sighted on this task contrasts with the results of old studies on the recognition of geometric shapes [30]–[33] in which the authors observed no notable difference between early blind and blindfolded sighted. This difference could be explained by the fact that congenitally blind people benefit from a higher haptic experience (or training) than the blindfolded sighted and that they have much more skills at recognizing haptically a great variety of shapes. Though the manual exploratory analysis reveals that the congenitally blind and the blindfolded sighted mostly used the “metric process” to recognize geometric shapes, the two groups did not use the different exploratory procedures in the same manner. So while the blindfolded sighted used almost exclusively metric procedure, the congenitally blind used the three exploratory procedures in a more inconstant way (71.4% metric procedure, 10.85% of contour following, 11.38% of enclosure). Moreover the main exploratory procedure used by the two groups was more efficient in the congenitally blind than in the blindfolded sighted. This better recognition of the correct shapes seems to be due to a more appropriate choice of exploratory procedures and to a better efficiency of these procedures. However, it should be noted that tough the congenitally blind obtained better recognition rates than blindfolded sighted, no differences appeared between the two groups on recognition time.

In our study, the prototype effect was observed in the haptic modality only on recognition times and when participants benefited from visual experience. This result contrasts the findings of Woods et al. (2008) [16] showing that the canonical views of objects (prototypes) would promote the recognition rate of these objects in haptics. It could be argued that the haptic spatial functioning could have been influenced by visual representations because of the dominant function of vision in human spatial processing [for reviews 34–39]. Visual recoding of haptic spatial information was demonstrated in shape recognition and reconstruction [33], in spatial localization [40], in the vertical-horizontal illusion [38], [41], in the haptic judgment of orientation [42], in tactual picture identification [43], and in the production of drawings of objects at a slant [43]. During recognition, the blindfolded sighted seemed to match the perceived stimulus with the stored view of this stimulus in memory and were more efficient when the perceived stimulus was very similar to the stored representation [44]–[45]. This is in line with the visual mediation model proposed by Lederman et al. (1990) [20] (see introduction section). Otherwise, it was possible that the recognition of shapes with axes of symmetry aligned with a framework is easier.

Our findings suggest that the prototype effect is not intrinsic to the haptic modality but depends on visual experience. Since prototypical shape and non prototypical shape differed mainly in their orientation, our results were in line with those of Gori et al. (2010) [46] showing that haptic orientation discrimination was impaired in blind children and suggesting a principal role of vision in haptic orientation perception. Furthermore, the occurrence of visual and haptic prototypical shapes in the recognition of geometrical shape seems to depend on visual exposure to these prototypical shapes existing in our environment. Indeed, the fact that the congenitally blind were not more efficient on the prototypical shapes can be explained by the fact that during the school period, training in geometry is impaired because of the practical difficulty to produce raised geometrical figures adapted to haptics. Therefore, congenitally blind children and adolescents may be less often exposed to the canonical geometrical shapes found in geometry books or produced on the blackboard by the sighted teacher.

Finally, though our findings provide new support for understanding how objects are represented in memory and subsequently recognized by the sighted, results do not allow us to determine whether the internal representations of geometric categories of congenitally blind differ from those of the sighted. Indeed, Woods et al. (2008) [16] showed that the prototype of familiar and unfamiliar objects observed in haptic modality differed from those classically reported in visual perception. It was therefore possible that the geometric shapes categories were structured around a prototype in the congenitally blind but that this prototype did not match the visual prototype. Drawings provide a good tool to investigate the nature of internal representations and their development [1], [47]–[50]. It would be interesting in a further study, to propose to the congenitally blind a drawing production task of geometric shapes in order to investigate the organization of geometric shape categories in people who do not benefit from visual experience.

## Supporting Information

Experiment S1 **The visual recognition of geometrical shapes in adolescents.**
(DOC)
