# Supplementary material for: The Haptic Recognition of Geometrical Shapes in Congenitally Blind and Blindfolded Adolescents: Is There a Haptic Prototype Effect?
Source: PLoS One. 2012 Jun 28;7(6):e40251. doi: 10.1371/journal.pone.0040251 (PMC3386238; doi:10.1371/journal.pone.0040251)
Supplement: Experiment S1 — The visual recognition of geometrical shapes in adolescents. (DOC) [file pone.0040251.s001.doc]

**Supporting Information**

Experiment S1: The visual recognition of geometrical shapes in adolescents

The main goal of this control experiment was to confirm the existence of “the prototype effect” (better recognition of the prototypical shapes compared to the non prototypical one) in the sighted adolescents with visual stimuli similar to those of our main experiment. Twelve young sighted (boys) aged 15.5 years on average (SD = 0.68) schooled in a French technical high school participated in this study. The participants were matched on age, schooling and socio-economic level with the blindfolded sighted group.

The same stimuli (square, rectangle and triangle) as in main experiment were transposed into a visual format and were presented to subjects on a computer screen. All target shapes and distractors were presented in small and large size. The large shapes have a size of 244 x 244 pixels for the square, 339 x 244 pixels for the rectangle and 281 x 244 pixels for the triangle. The small shapes have a size of 155 x 155 pixels for the square, 215 x 155 for the rectangle and 155 x 136 pixels for the triangle. The shapes were filled black and presented on the center of a white background. For each category of geometric shapes, targets and distractors (small and large) were presented in a canonical way (i.e. the shape was presented standing on its base), called « prototypical », or in an oriented way (i.e. the canonical shape was rotated from 45 degrees), called « non prototypical ». In total, thirty-six stimuli were presented to the subjects (Figure 1).

Each participant was asked to recognize a geometrical shape presented on a computer screen. Before each stimulus, the name of a shape category (square, rectangle, triangle) written in black capital letters was presented at the center of a white background. For example, all stimuli belonging to the square category were preceded by a screen on which was written SQUARE. The same procedure is applied for the rectangle and triangle categories. Participants had to decide as quickly as possible whether the shape stimulus corresponds to the name of the geometric shape. The V button of the keyboard correspond to the answer “yes” and the N button correspond to the “no” answer. This double choice task was preferred to a faithful replication of our main experience where participants would have had the choice between 4 response buttons. The thirty six stimuli are randomly presented. The recognition time (milliseconds) and the nature of responses (correct or false recognition) are recorded.

The analysis of the recognition rate showed that the target shapes (square, rectangle, triangle) are correctly identified by the sighted with a recognition rate of 85.93 % (SD=10.36), with 11.25 % of false recognitions (SD = 7.11). In order to examine the “prototype effect”, we used Student test on recognition rate and on recognition time in order to compare the responses observed for the prototypical target and for the non prototypical target. Regarding the recognition rate in %, the results showed that the prototypical targets are better recognized (M = 93.05 and SD = 11.1) than the non prototypical targets (M = 75 and SD = 20.7; t(11) = 2,39 ; p<.05). Regarding the recognition time in ms, a prototypical effect was observed. Thus, the prototypical shapes (Mean = 765.77 and SD = 241.36) are faster recognized than the non prototypical target (Mean = 1037.23 and SD = 283.41; t(11) = 4.58; p <.01).

The analyses of recognition rate and recognition time showed that the prototypical target shapes were better recognized than the non prototypical target shapes. This control study confirmed with other measures (reaction time) in sighted adolescents the results observed in children by Pinet and Gentaz (2008) [6] and showed the relevance of the set of stimuli used in the main experiment to examine the haptic prototypical effect in congenitally blind people.
